# Supplementary material for: The causal relationship between the human gut microbiota and pyogenic arthritis: a Mendelian randomization study
Source: Front Cell Infect Microbiol. 2024 Nov 26;14:1452480. doi: 10.3389/fcimb.2024.1452480 (PMC11629706; doi:10.3389/fcimb.2024.1452480)
Supplement: Supplementary file 1 [file DataSheet1.zip › mendelian test/ebi-a-GCST90027533.csv_forest.pdf]

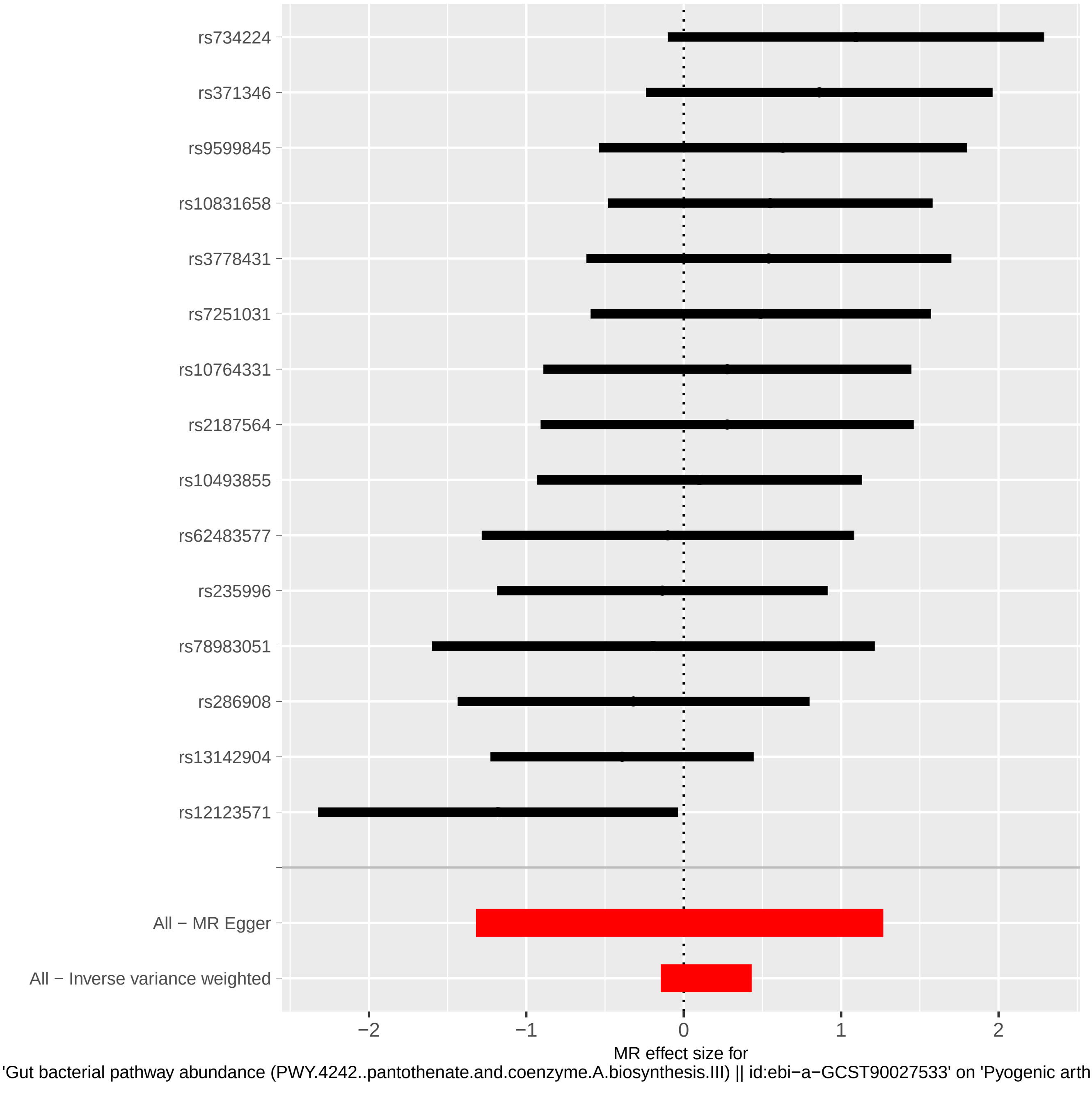
rs734224
rs371346
rs9599845
rs10831658
rs3778431
rs7251031
rs10764331
rs2187564
rs10493855
rs62483577
rs235996
rs78983051
rs286908
rs13142904
rs12123571
All − MR Egger
All − Inverse variance weighted
−2
−1
0
1
2
MR effect size for
'Gut bacterial pathway abundance (PWY.4242..pantothenate.and.coenzyme.A.biosynthesis.III) || id:ebi−a−GCST90027533' on 'Pyogenic arth
